# Smoking cessation with smartphone applications (SWAPP): study protocol for a randomized controlled trial

**DOI:** 10.1186/s12889-019-7723-z

**Published:** 2019-10-29

**Authors:** Janina Lüscher, Corina Berli, Philipp Schwaninger, Urte Scholz

**Affiliations:** 1 Applied Social and Health Psychology, Department of Psychology, University of Zurich, Binzmuehlestrasse 14 / Box 14, 8050 Zurich, Switzerland; 2 Applied Social and Health Psychology and University Research Priority Program “Dynamics of Healthy Aging”, Department of Psychology, University of Zurich, Binzmuehlestrasse 14 / Box 14, 8050 Zurich, Switzerland

**Keywords:** Smoking cessation, Randomized controlled trial, Social support, Smartphone applications, Buddy, Buddy support, Everyday life, Smokerlyzer

## Abstract

**Background:**

Tobacco smoking remains one of the biggest public health threats. Smartphone apps offer new promising opportunities for supporting smoking cessation in real-time. The social context of smokers has, however, been neglected in smartphone apps promoting smoking cessation. This randomized controlled trial investigates the effectiveness of a smartphone app in which smokers quit smoking with the help of a social network member.

**Methods:**

This protocol describes the design of a single-blind, two-arm, parallel-group, intensive longitudinal randomized controlled trial. Participants of this study are adult smokers who smoke at least one cigarette per day and intend to quit smoking at a self-set quit date. Blocking as means of group-balanced randomization is used to allocate participants to intervention or control conditions. Both intervention and control group use a smartphone-compatible device for measuring their daily smoking behavior objectively via exhaled carbon monoxide. In addition, the intervention group is instructed to use the SmokeFree Buddy app, a multicomponent app that also facilitates smoking-cessation specific social support from a buddy over a smartphone application. All participants fill out a baseline diary for three consecutive days and are invited to the lab for a background assessment. They subsequently participate in an end-of-day diary phase from 7 days before and until 20 days after a self-set quit date. Six months after the self-set quit date a follow-up diary for three consecutive days takes place. The primary outcome measures are daily self-reported and objectively-assessed smoking abstinence and secondary outcome measures are daily self-reported number of cigarettes smoked.

**Discussion:**

This is the first study examining the effectiveness of a smoking cessation mobile intervention using the SmokeFree Buddy app compared to a control group in a real-life setting around a self-set quit date using a portable objective measure to assess smoking abstinence. Opportunities and challenges with running studies with smoking participants and certain design-related decisions are discussed.

**Trial registration:**

This trial was prospectively registered on 04/04/2018 at ISRCTNregistry: ISRCTN11154315.

## Background

Smoking remains one of the leading preventable causes of premature death worldwide [1]. Smokers who smoke approximately 16 cigarettes per day lose about 11 min of their lifetime per cigarette smoked [2]. Additionally, smoking is an important risk factor for serious health problems and life-threatening diseases such as lung cancer, coronary heart disease and stroke [1]. Despite the knowledge about the negative consequences of smoking, in 2016 every fourth person aged 15 and older smoked in Switzerland [3]. High relapse rates indicate that quitting smoking is a major challenge [4]. The core of relapse prevention is to strengthen the capability to manage high-risk situations (e.g., cue-induced cravings [5, 6]). Although many evidence-based smoking cessation interventions exist (e.g., behavioral counselling, medications, nicotine replacement therapy [7]), the majority of smokers quit unassisted without the help of pharmacological aids or other interventions [8, 9]. The accessibility and availability of mobile technology (e.g., mobile phones/smartphones) offers new promising opportunities for cost-effective interventions in everyday life [10]. As most relapses occur within the first weeks after the quit attempt [4, 11], such interventions have the potential to deliver support when it is most needed [11, 12].

### Smartphone apps to promote smoking cessation

In 2016 mobile-broadband networks reached 84% of the global population [13]. Among a sample of US and UK smokers the prevalence of smartphone ownership with internet access with over 75% was high [14]. In the context of smoking cessation, there are already numerous studies on text messages based interventions using mobile phones [15]. The efficacy of these interventions is tentatively confirmed as positive effects were found up to 6 months after quitting smoking [16, 17]. In contrast, there are far fewer smartphone app-based smoking cessation intervention studies in daily life [18]. Due to the high prevalence of smartphone usage, delivering health promotion interventions using smartphone apps is a promising approach, especially because of the proximity to users, cost effectiveness, location independence, possibility of tailoring and providing instant interactive support [19–21].

First studies on smartphone apps and smoking cessation provide good evidence that apps are a useful tool to promote the implementation of the intended behavior (c.f., [10, 22]). For example, preliminary results of the SmokeFree28 app showed higher cessation rates for 28 days than unaided cessation [23]. Also, in a RCT of the smartphone app REQ-Mobile smokers who used the app showed higher abstinence rates at 30 days compared to smokers who did not use the app [10]. Furthermore, in another RCT smokers who received a smartphone app to quit were more likely to be continuously abstinent at 1 month, 3 months and 6 months after quitting [22]. All those apps assessed smoking abstinence by self-report only.

In a content analysis of smartphone apps for smoking cessation, calculator apps were the most common category (38.8% of all apps), followed by hypnosis apps (17.3%), rationing apps (15.3%), trackers (12.2%), informational apps (6.1%), games (3.1%) and lung health testers (2.0%) [20]. In line with these findings, another content analysis of Android smoking cessation apps also found that apps predominantly provide simple tools, as for example calculators, calendars, trackers or distractors [24]. Most available apps used very little evidence-based content to support quit attempts [19]. In approximately 55% (*n* = 75) of the apps no behavior change techniques (BCTs [25];) were present [19]. Apps rarely referenced smokers outside of the app to a quit helpline or provided opportunities to reach out for social support from a social network member [20]. Of 225 rated Android apps only 6 (2.7%) had also content regarding social support for smokers [24]. However, one of the key recommendation of the clinical practice guideline Treating Tobacco Use and Dependence [7] is to deliver social support in individual, group or telephone counseling settings. External resources such as social support seem promising in helping smokers to quit and might buffer the daily stress smokers experience while quitting [4, 11, 26].

### Social support and smoking cessation

Social support comprises resources provided to a person in need and can include the following functions: emotional (e.g., comforting, encouraging) and instrumental (practical help or assistance) [27, 28]. It can be distinguished into a recipient’s retrospective report of support received (i.e., *received* support), a provider’s retrospective report of support given (i.e., *provided* support), and the perceived prospective potential access to social support resources (i.e., *perceived support*) [27–29]. There is evidence from longitudinal, prospective studies that higher received social support from a network member is related to higher abstinence rates (e.g., [30]). In a recent intensive longitudinal mobile phone-based study with single-smoker couples the fine-grained temporal dynamics of daily social support and daily number of cigarettes smoked was investigated in the process of quitting [26]. Increases in received emotional and instrumental support were related to less smoking, and effects were more pronounced after a self-set quit date when support is most needed. Also, support provided from the non-smoking romantic partner was associated with less smoking [26]. Consistent with these findings, in another intensive longitudinal mobile phone-based study with dual-smoker couples, received emotional and instrumental support was related to less smoking after a joint self-set quit date [31]. For men only, female partner’s provided emotional and instrumental support was also related to fewer cigarettes smoked in dual-smoker couples [31].

Up to date, several intervention studies exist to foster social support [32]. However, results on the effectiveness are mixed [33, 34]. In a review investigating RCTs that compared smokers who received an intervention to enhance peer or partner support with smokers who did not receive the support intervention, the effectiveness of the social support interventions was not clearly given [35]. In another review there was also lack of evidence regarding the efficacy of the use of buddies in community interventions [11]. For example, in a study examining the effectiveness of a social support intervention with a buddy in a group treatment program to aid smoking cessation, smokers in the buddy condition (smokers were paired with another participant to provide mutual support) were no more likely than smokers in the control condition to stay abstinent at one, four or 26 weeks [36]. Thus, it seems that buddy systems so far have been unable to improve abstinence rates of participants in group treatment programs.

Findings from intervention studies could, thus, not demonstrate the assumed effectiveness of partner or peer support on smoking cessation, supposably because these interventions were not successful in increasing social support in the first place [11, 37, 38]. One of the problems associated with this could be that these buddy systems often rely on unacquainted buddies, not persons from one’s own personal social network. Moreover, buddies need instructions on how to support smokers during a quit attempt to ensure high quality of social support. The ideal timing of support seems relevant; that is, when in the process of smoking cessation social support is most helpful for smokers [11, 39]. This emphasizes that future intervention studies enhancing social support should take place in smoker’s everyday life and social support should be available directly when smokers need it. This issue can be addressed by applying ecological momentary interventions (EMI [40];) that are characterized by the delivery to people as they go about their everyday lives in combination with momentary ambulatory assessments [40]. EMI’s can be used to stimulate processes that take place in everyday life, as for example social support. Thus, a smartphone app for targeting social support in the context of smoking cessation would allow not only to address the aspect of timing but could also provide the target person with tailored social support while quitting. There is first evidence from a randomized pilot trial comparing automated text-messaging support (control group) with automated text-messaging support plus personalized texts from a peer mentor who formerly smoked (peer-mentor group) that smoking abstinence at 3 months was higher for the group with peer mentors compared to the control group [41]. However, the role of social support from a network member via a smartphone app has not yet been investigated in the context of smoking cessation. The present study investigates the efficacy of such a smartphone app, the SmokeFree Buddy app, connecting a smoker with a freely chosen support buddy to promote smoking cessation in smoker’s everyday life using a randomized controlled trial (RCT).

### The SmokeFree buddy app

As part of the tobacco prevention campaign in Switzerland the Federal Office of Public Health (FOPH) and its partners developed a smartphone app –the SmokeFree Buddy app - to encourage smokers’ intention to quit and to offer social support interactively from a self-chosen person (buddy) while quitting smoking. The SmokeFree Buddy app was developed from experts based on the empirical and theoretical evidence of social support from a social network member for smoking cessation. Mobile interventions have the potential to intervene at any time, in a tailored manner and during actual experiences in people’s everyday life [40]. The SmokeFree Buddy app, thus, aims at enabling smokers to quit with the help of a self-chosen buddy from one’s social network and offers the possibility of enhancing social support resources and availability directly after a self-set quit attempt in smoker’s everyday life. The buddy then gets instructed via the smartphone app on how to support the smoker during his/her quit attempt. In addition to self-reported smoking behavior the present RCT uses an objective device to assess smoking abstinence via a “smokerlyzer” with a corresponding smartphone app and is therefore, the first study assessing smoking abstinence objectively on a daily basis.

A general criticism on standard RCT’s is that the level of longitudinal assessment is usually on a macro-time level (e.g., baseline and a 1 month follow-up). This is also the case for intervention studies on social support in the context of smoking cessation (i.e., [11, 37, 38]). However, relatively little is known on when an intervention reaches its effect and what time window would be appropriate to capture it. As such, the choice for the follow-up time points is often made on an arbitrary basis. By using micro-time assessments (e.g., daily) during an ongoing intervention, it is possible to answer questions on how an intervention effect unfolds over time, when it reaches its maximal effect and whether it is maintained or levels off quickly [42]. Moreover, it allows investigating how people respond differently to the intervention. In the present study, we combine micro-time and macro-time assessments by using an intensive longitudinal intervention design with three end-of-day diary periods across 6 months. The 6 months time period was chosen for better comparability with previous studies using a six-month follow-up.

### Aims of the present study

This study aims to test the effectiveness of a smoking cessation mobile intervention using the SmokeFree Buddy app compared to a control group in a real-life setting around a self-set quit date, and to examine the hypothesized mediating mechanisms. More specifically, the research aims are the following: 1) Is the SmokeFree Buddy app an effective intervention to promote daily abstinence rates and to reduce daily number of cigarettes smoked in adult smokers at the self-set quit date, 3 weeks (end of intervention) and 6 months later in comparison to a control group? The primary outcome measures are daily self-reports of smoking abstinence (subjective) and daily smoking abstinence using exhaled carbon monoxide (objective). Daily self-report of number of cigarettes smoked serves as secondary outcome measure. 2) What are the trajectories over time of daily smoking abstinence rates and daily number of cigarettes smoked in the intervention compared to the control group? 3) Does the SmokeFree Buddy app increase social support and self-regulatory processes in daily life and do these constructs serve as mediating mechanisms explaining the effect of the SmokeFree Buddy app? Hypothesized mediators are daily self-reports of intensity and quality of social support, self-efficacy and action control. For the specific hypotheses please see the trial registration: ISRCTN11154315 (10.1186/ISRCTN11154315). This trial was prospectively registered on 04/04/2018.

## Methods/design

**Fig. 1:**
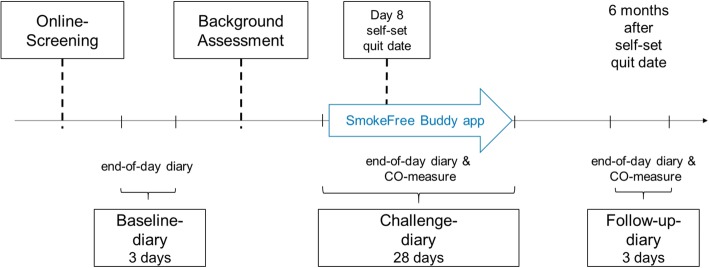
The longitudinal study design

This single-blind, two-arm, parallel-group, randomized controlled trial comprises an intensive longitudinal design with three end-of-day diary periods: A *baseline diary* (3 consecutive days), a *challenge diary* from 7 days before the self-set quit date, on the self-set quit date until 20 days after the self-set quit date (28 consecutive days) and a *follow-up diary* 6 months after the self-set quit date (3 consecutive days). See Fig. 1 for the longitudinal design.

Participants of this randomized controlled trial are adult smokers, who smoke at least one cigarette daily [43], intend to quit smoking during the study, and own a smartphone with access to mobile internet. Moreover, participants should speak German fluently, should not work in 24-h shifts, should not participate in a professional smoking cessation program, and should not already use a smoking cessation smartphone app. Recruitment takes place in Switzerland and is organized via advertisements in newspapers, online platforms and on webpages, flyers and postings in the university, medical facilities and local companies. Interested individuals can complete a prescreening assessment online, for which they provide informed consent and that checks inclusion and exclusion criteria. In case smokers meet all inclusion criteria, they are contacted via email and sent the link for an online end-of-day diary questionnaire for three consecutive days (baseline diary).

After completing the baseline diary participants are contacted and invited to the lab for a background assessment. At the background assessment participants first receive full information on the study, and provide written informed consent. After completing a comprehensive background questionnaire, participants are asked to set a self-set quit date within the next 6 weeks. They are instructed to fill in the online challenge diary from 7 days before the self-set quit date, on the self-set quit date and 20 days after the self-set quit date. During those 28 days all participants receive a daily text message with the link for the questionnaire at seven o’clock in the evening. Subsequently, participants receive an instruction for the personal mobile objective smoking abstinence measure, the iCO Smokerlyzer (Bedfont Scientific Ltd.), and the corresponding smartphone app to measure the exhaled carbon monoxide (CO) daily. A first measurement of CO with the iCO Smokerlyzer is already taken in the lab. Lastly, participants of the intervention group are introduced to the smartphone app (for details on randomization procedure and the intervention see below). Six months after the self-set quit date, participants of both groups are contacted via email and asked to participate in the follow-up diary phase for completing online end-of-day diaries for three consecutive days and daily objective assessments of smoking with the iCO Smokerlyzer.

As a main incentive for study participation, all participants will receive the iCO Smokerlyzer a carbon monoxide monitor for the smartphone, with a value of 60 CHF (= 63 USD). Additionally, with completion of the six-month follow-up, participants from both groups are given entry into a lottery. The lottery has one main prize of 200 CHF (= 209 USD), and 40 prizes with a value of a 50 CHF (= 52 USD) shopping vouchers. Participants in the role of the supporting buddy will be reimbursed with 50 CHF (= 52 USD) with completion of the study.

### Randomization

The randomization comprises a random assignment to one of the two groups (intervention group and control group). Blocking as means of restricted randomization is used: To meet balance and predictability criteria [44, 45] variable blocks (4, 6, 8) are used. Within a block of participants, 50% are assigned to the intervention group and the control group. Before the beginning of the study, a computerized random-number generator (http://www.randomization.com) is used for sequence generation of blocks. This allocation sequence is generated by an assistant not part of the study team and to ensure allocation concealment concealed in a set of sealed, numbered envelopes until group assignment. On the day of the background assessment in the lab, the interviewer conducting the session opens the appropriate numbered envelope and prepares the study material accordingly. Throughout the study, participants are blinded to the group assignment (i.e., single-blind RCT).

### Detailed description of intervention procedure and control group

This study has an EMI design [40] and comprises two groups: a SmokeFree Buddy app intervention group and a control group (see below). As described above, at the background assessment all participants are instructed to set a self-set quit date, to fill out the end-of-day diaries for 28 consecutive days and to measure the exhaled carbon monoxide daily during the challenge diary phase using the iCO Smokerlyzer. Setting a self-set quit date comprises the BCT “goal setting of behavior” [25]. The daily CO measure comprises the BCT “feedback on behavior” and “self-monitoring of behavior” [25]. For an overview of the most prominent BCTs included in the study please see Table 1.

**Table 1:** Overview of intervention components and corresponding most prominent BCTs in intervention group (IG) and control group (CG)

Groups	Intervention components	Behavior change techniques (BCTs)
IG + CG	Set a self-set quit date	Goal setting of behavior
IG + CG	Daily CO measure and end-of-day diary across 28 days	Feedback on behavior
		Self-monitoring of behavior
IG only	The SmokeFree Buddy app	Social support (emotional), social support (practical)
		Social reward
		Feedback on behavior
		Self-monitoring of behavior, others monitoring with awareness
		Discrepancy between current behavior and goal standard
		Non-specific reward
		Information about health consequences

*IG* Intervention Group, *CG* Control Group

#### Intervention group: SmokeFree buddy app group

In addition to the instructions of the daily assessments with the iCO Smokerlyzer, target persons of the intervention group will be introduced to the SmokeFree Buddy app and instructed on how to use it during the challenge diary phase (7 days before the self-set quit date, on the self-set quit date until 20 days after the self-set quit date). The SmokeFree Buddy app is a smartphone app that aims at enabling smokers to quit with the help of a self-chosen buddy from one’s social network and offers the possibility of enhancing social support resources and availability directly after a self-set quit attempt in smoker’s everyday life.

Participants of the intervention group have to identify a personal buddy (self-chosen from their personal social network) with whom they will start the smokefree challenge. The smartphone app has a scientific background, and was developed by the Federal Institute of Health in Switzerland in collaboration with the Institut de Santé Globale of the University of Geneva in Switzerland. For more details about the SmokeFree Buddy app please see https://www.smokefree.ch/en/buddy-app/. For an overview of the most prominent BCTs of the SmokeFree Buddy app, please see Table 1.

The SmokeFree Buddy app has the following features: (1) Buddy support. The main feature of the app is a chat function through which the smoker and the personal buddy are connected. The chat is the app’s direct communication channel for all text-messages between the smoker and the buddy. Apart from the text-messages sent, the chat lists all events occurring within the app (e.g. notifications, mood change, bonuses; see below). The app informs the buddy for example about the smoker’s current mood or craving and provides them with the option of reacting immediately, e.g. with a supportive message. The buddy can choose one of many preset messages, which can be customized or supplemented with a personal comment. Unlike anonymous computerized notifications, support through the SmokeFree Buddy app is personal, from a buddy and can be tailored to the smoker’s need. (2) Current mood and notification buttons. The app provides a feature to indicate the current mood state and intensity of the smoker (e.g. joy, depressed, anger) that is also visible for the buddy. Additionally, there are three different notification buttons on the smoker’s side: SOS! (sending an emergency message), desire (communicating an urge/craving to smoke, from 1 [= slight] to 5 [= strong]), and lapse (number of cigarettes smoked). A change in mood or a click on one of the notification buttons directly sends a message to the buddy (via chat), requesting their support as soon as possible. The app provides background information of these different situations and preset messages for the buddy, which can be personalized. (3) Bonuses. With the bonus-feature the buddy can motivate the smoker. Various bonuses are available such as encouraging, confirmation of success or advice for a particular challenge. In the bonus gallery the target persons are able to see the trophies that they have earned any time. (4) Performance statistics. These statistics show the target person’s achievement to date: The number of smoke-free days, the number of cigarettes that the smoker did not smoke (i.e. being smoke-free) and the amount of money he or she was able to save. (5) Knowledge base. A Knowledge base allows smoker and buddy to rapidly retrieve information on smoking and quitting and share it with each other via the chat. There is also a permanent information bar that shows health benefits of stopping smoking over the course of time. Moreover, the app provides contact information to a quit helpline and professional smoking cessation counseling.

After being introduced to the app, participants of the intervention group are instructed to find a buddy with whom they will communicate through the app during their self-set quit attempt. The buddy has to be a non-smoker for at least 6 months [46]. Furthermore, they should also own a smartphone with access to mobile internet, should speak German fluently and should not work in 24 h shift work. The supporting buddy of smokers in the intervention group receives an email with a link to a background questionnaire that checks for eligibility criteria, asks for informed consent, and contains instructions on how to use the app. Moreover, buddies are instructed to also fill out an end-of-day diary from 7 days before the quit date of the smokers, the quit date itself, and 20 days after (challenge diary phase). One day prior to the first diary day, smokers and buddies receive a reminder on how to connect and to use the SmokeFree Buddy app.

#### Control group

The participants in the control group will also announce a self-set quit date, measure the exhaled carbon monoxide daily using the iCO Smokerlyzer and fill out the end-of-day diary for 28 consecutive days during the diary phase. Smokers in the control group will, thus, have the same setting as the intervention group only without the SmokeFree Buddy app.

### Measures

#### Smokers in the intervention and control group

The prescreening questionnaire assesses inclusion and exclusion criteria as well as measures on socio-demographics and habitual smoking behavior adapted from Keller et al. [47]. At the background assessment smokers complete a comprehensive questionnaire on current smoking, smoking history, self-regulation (e.g., smoking-specific self-efficacy, action planning, action control) [48, 49], health status [50], personality and further potential control variables. Additionally, an objective baseline measure for smoking status is taken using the mobile iCO Smokerlyzer. All three end-of-day diary phases (baseline diary, challenge diary and follow-up diary) include short scales or single items on self-reported daily smoking abstinence, number of cigarettes smoked, quantity and quality of received social support from the buddy and from other network members [26, 31, 51], quantity and quality of received social control from the buddy and from other network members [52–54], self-regulation (e.g., self-efficacy, action planning, action control) [49], indicators of subjective well-being [55], relationship quality [56] and further potential control variables. Smokers in the control group do not receive items relating to the buddy. Smokers in the intervention group additionally report on the use of the SmokeFree Buddy app. On the first day of the follow-up diary, smokers are asked to report on their smoking abstinence and lapses since their self-set quit date. During both the 28-days challenge diary and the 3-day follow up diary, all participants measure their daily smoking behavior objectively with their personal mobile iCO Smokerlyzer.

#### Buddies of smokers in the intervention group

Buddies complete a combined prescreening and background questionnaire, assessing inclusion and exclusion criteria, socio-demographic variables, motivation to assist the smoker, health status [50], personality, and further control variables. They only participate in the 28-day challenge diary phase that contains relatively parallel items to the diary of the smokers assessing the buddies’ subjective well-being [55], quantity and quality of provided social support [26, 31, 51], quantity and quality of provided social control [53, 54], relationship quality [56], as well as their perspective on the smoker’s regulation during the quit attempt.

### Statistical analysis

Upon completion of data collection, data will be cleaned and prepared for data analyses. Primary and secondary outcome variables and mediating mechanisms will be checked for distribution, outliers and missing patterns, and appropriate steps will be taken where necessary (cf., [57]). All analyses follow intention-to-treat principles, taking advantage of all available data points. Low levels of missings will be expected, with previous studies showing high completion rates (> 90%, e.g., [26]). Intermittent missing data will be handled by maximum likelihood (MI) estimation. Participants who completely drop out of the study will be treated as smokers. Preliminary analysis will include a randomization check to test for baseline differences between intervention and control participants. For the main data analysis, we will use multilevel modeling to account for the nested structure of daily measures within individuals [58]. To examine the first research question on the effects of the intervention using the daily diary, a set of generalized linear mixed models will be performed in SPSS and R. Daily smoking abstinence will be modeled as a function of time group and group by time, testing group differences at the quit date (model 1) and 3 weeks later (model 2) by centering the time variable accordingly. Model 3 will separately test group differences 6 months later. For the primary outcomes of subjectively and objectively reported smoking abstinence (dichotomous), we will use mixed logistic regression analyses. For the secondary outcome of subjectively reported number of cigarettes smoked, a count variable, we will use a negative binomial model with zero inflation [59, 60]. To test the third research question on the mediating mechanisms of the intervention effect, we will use a multilevel approach to mediation analysis based on multilevel structural equation models [61, 62].

### Power analysis

To secure adequate power for the main analysis of the intervention effect on smoking abstinence at the quit date, 3 weeks and 6 months later, we performed a priori sample size calculations using the G*Power program [63]. Based on a power of 0.80 and a two-tailed Type 1 error probability of .05, a total sample size of *N* = 128 is suitable to detect a 24% difference in smoking abstinence for the intervention group, drawing on meta-analyses of mobile-phone interventions on short and long-term smoking abstinence (RR’s between 1.7 and 2.1 [16, 17];). We assumed an abstinence rate of 27% in the control group 6 months after the self-set quit date, based on own previous studies using a highly comparable, albeit correlational, design with daily diaries around a self-set quit date in smokers and their romantic partners [64, 65]. Moreover, attrition rates between 20% (e.g., [31]) 6 months after the self-set quit date, or below 30% [15] have been reported. Thus, assuming an attrition rate of 25%, the targeted sample size is *N* = 160 smokers (*n* = 80 per group) and an additional *N* = 80 buddies.

### Ethics

The study was approved by the Ethics Committee of the Faculty of Arts and Social Sciences of the University of Zurich, 13 December 2017 (Reference number: 17. 12. 13).

## Discussion

Quitting smoking or the reduction of the smoking behavior can have important health advantages [1]. Quitting smoking is, however, very challenging as shown by low long-term abstinence rates [4]. Consequently, successful smoking cessation interventions are of key importance. Research on social support demonstrates the importance of social network members with regard to smoking cessation in smoker’s everyday life [11, 26, 31]. However, up to date intervention studies on social support and smoking cessation have not been very effective [11, 37, 38]. One explanation is that timing of social support transactions have not been considered and that social support was not tailored to the needs of the target person [39], thus, the interventions did not manage to effectively increase social support.

Smartphone apps to promote smoking cessation via social support from a social network member in smoker’s everyday life seem promising to address this research gap. This study aims to test the efficacy of the SmokeFree Buddy app, which aside from other BCTs primarily aims at enhancing social support resources by involving a supporting buddy in the quit attempt. We test whether the use of the SmokeFree Buddy app is more effective for smoking cessation over and above monitoring one’s smoking behavior daily. The study applies an EMI design [40] with micro-time assessments to investigate direct intervention effects on postulated mediating mechanisms and smoking outcomes on a daily basis. Because the app allows tailoring the support to the needs of the smoker, we hypothesize that not only the amount of support (i.e., quantity), but also the quality of support can be enhanced, e.g., that smokers report the support received to be helpful. Previous reviews have identified the importance of the quality of support for support outcomes, that is not only whether but also how support is provided (cf., [66]). For example, support should be provided responsively to recipients’ needs (e.g., [67]) and sensitively. This is likely to also be highly relevant for aiding smoking cessation. The present study thus aims to shed light on how helpful support can be facilitated and how it relates to successful smoking cessation. Moreover, using a daily objective measure to assess smoking abstinence via iCO Smokerlyzer and the corresponding smartphone app in both groups, we further hypothesize that additional self-regulation variables such as self-efficacy and action control (i.e., continuously monitoring and evaluating an ongoing behavior with regard to one’s standards [68];) are increased, albeit for both groups. Evidence from a meta-analysis suggests that interventions using behavior change techniques such as self-monitoring, along with the use of social support were most effective for smoking cessation in people with chronic obstructive pulmonary disease [69].

Following we will discuss some challenges regarding this study’s procedure. The procedure for the intervention group is very complex and complicated. Participants of the intervention group have to find a non-smoking buddy who supports them during their quit attempt and who is also willing to participate in the study. Moreover, they have to install the app and invite the buddy on their own at home to do so as well. Another aspect is that we let the participants use their own smartphones. We unfortunately have to exclude everyone who does not have an own smartphone or a mobile internet access on their own smartphone. However, results of a representative report showed that 100% of adolescents and their families have at least one smartphone at home and thus, coverage of smartphones in Switzerland is very high [70]. At the same time, our population of interest are individuals that can potentially use the app. Using one’s own smartphone in everyday life moreover has the advantage of ensuring a relatively natural setting for the intervention, hence, increasing ecological validity. Furthermore, we are not able to control the intervention fidelity in terms of using the mobile application if participants will not give us permission to access and use the usage data or if participants will use other channels like text messages to chat about the smoking cessation instead of the SmokeFree Buddy app. Finally, there is a trade-off between assessing abstinence rates with the iCO Smokerlyzer, a state-of-the-art objective ambulatory assessment measure, and its antecedents by self-report. Moreover, there exists the possibility of confounding effects of the ambulatory assessment with the buddy social support intervention. Reporting on their own smoking and measuring the smoking behavior also objectively with their own smartphone can be in itself a self-monitoring intervention for the participating smokers. However, this effect of the self-reports and the objective measure of CO is constant between the intervention and the control group and we are especially interested in the effect of the SmokeFree Buddy app over and above the self-monitoring component. Another important point concerns the successful recruitment of smokers. Besides that smokers need to be willing to participate in an intensive longitudinal study, smokers participating in the present study have to meet many inclusion criteria such as smoking at least one cigarette per day, having the intention to quit smoking etc. Therefore, the recruitment process can be very challenging as already experienced by researchers investigating smokers’ everyday life in an intensive longitudinal design (c.f, [26, 31]).

This study will despite these challenges substantially expand our knowledge with regard to the effectiveness of a smoking cessation mobile intervention in a real-life setting around a self-set quit date.

## Data Availability

Not applicable.

## Abbreviations

App: Application
BCT: Behavior change technique
CO: Carbon monoxide
EMI: Ecological momentary intervention
RCT: Randomized controlled trial
